# Soft Alkyl Ether Prodrugs of a Model Phenolic Drug: The Effect of Incorporation of Ethyleneoxy Groups on Transdermal Delivery

**DOI:** 10.3390/molecules14104231

**Published:** 2009-10-22

**Authors:** Joshua Denver Thomas, Susruta Majumdar, Kenneth Berry Sloan

**Affiliations:** 1 Laboratory of Medicinal Chemistry, NCI, NIH, NCI – Frederick, Frederick, MD 21702, USA; 2 Laboratory of Molecular Pharmacology and Chemistry, Memorial Sloan Kettering Cancer Center, 1275 York Ave, New York, NY 10021, USA; 3 Department of Medicinal Chemistry, University of Florida, P.O. Box 100485, Gainesville, FL 32610, USA

**Keywords:** topical delivery, Roberts-Sloan equation, water solubility, lipid solubility, prodrugs

## Abstract

Two different types of soft alkyl ether prodrugs incorporating ethyleneoxy groups into the promoiety have been synthesized for a model phenol (acetaminophen, APAP): alkyloxycarbonyloxymethyl type (AOCOM) and *N*-alkyl-*N*-alkyloxycarbonyl-aminomethyl type (NANAOCAM). The solubilities in isopropyl myristate, S_IPM_, and water, S_AQ_, partition coefficients between IPM and pH 4.0 buffer, K_IPM:4.0_, and the delivery of total species containing APAP through hairless mouse skin from IPM, J_MMIPM_, have been measured for the prodrugs. The J_MMIPM_ values were accurately predicted by the Roberts-Sloan (**RS**) equation. Only modest increases in J_MMIPM_ were realized (about 1.4 times) by each type. The only prodrug that was more water soluble and more lipid soluble than APAP did not improve J_MMIPM_ of APAP. This result may be due to the strong association of water molecules with the ethyleneoxy groups, and especially the triethyleneoxy derivative, which dramatically increases the molecular weight and depresses J_MMIPM_.

## 1. Introduction

Drugs containing a phenolic functional group constitute a large and important class of drugs. Unfortunately, phenols are good substrates for metabolic enzymes that conjugate and inactivate them during oral absorption [1]. Consequently, when confronted with a drug containing a phenol group, it may be useful to have the option of using a different route of delivery available. Topical delivery is one of those options. The major drawback for the topical delivery option is that many drugs do not have the prerequisite physicochemical properties necessary for their effective topical delivery. One way of overcoming the poor physicochemical properties of the drug is to make a prodrug.

Recent reports have established that a balance of lipid, S_LIPID_, and aqueous, S_AQ_, solubilities of the drug or prodrug, as well as small size, result in the optimization of drug or prodrug transdermal delivery regardless of the vehicle or membrane [2,3,4,5,6,7,8]. Based on Fick’s law for flux, J (Equation 1), the concentration of the drugs or prodrug in the first few layers of the membrane, C_M1_, provides the driving force for diffusion through the membrane. When a saturated solution of drug or prodrug in the vehicle, S_V_, is applied and is in equilibrium with the membrane, the driving force is the solubility of drug or prodrug in the first few layers of the membrane, S_M1_, and J becomes maximum flux, J_M_ (Equation 2). As long as the applied solution remains saturated, S_M1_ is maintained and the driving force is constant. Here D is the diffusion coefficient of the drug or prodrug in the membrane, L is the thickness of the membrane and the concentration in the last few layers of the membrane, C_Mn_, is assumed to be negligible. S_M1_ can be estimated from the product of S_V_ and the partition coefficient between the membrane and the vehicle, K_M1:V_. When the vehicle and membrane are both lipid-like materials as in this case (isopropyl myristate, IPM, and hairless mouse skin, respectively), K_M1:V_ can be calculated from K_M1:LIPID_ = K_M1:AQ_ /K_LIPID:AQ_. When (K_LIPID:AQ_)^y^c is substituted for K_M1:AQ_ and each K term is converted to its solubility terms on a log basis, log K_M1:LIPID_ is y log S_LIPID_ – y log S_AQ_ + log c – log S_LIPID_ + log S_AQ_. Substitution of log D_0_ – β MW for log D, collection of solubility terms and the constants (log D_0_/L and log c) into the new constant x and substitution of z for β gives equation 3: the Roberts-Sloan equation [4]:

J = (D/L) (C_M1_ – C_Mn_) (1)

J_M_ = (D/L) (S_M1_ – C_Mn_) (2)

log J_M_ = x + y log S_LIPID_ + (1 – y) log S_AQ_ – zMw (3)

A number of approaches to improving the balance of lipid and aqueous solubilities of a phenolic drug by making prodrugs to improve topical delivery have been reported. The first report was of alkylcarbonyl (AC) esters of morphine [9]. More recently AC [10], alkyloxycarbonyl, AOC [11] and alkylaminocarbonyl, AAC [12] derivatives of naltrexone have been reported to enhance its transdermal delivery. Another recent report [13] used AOC derivatives of acetaminophen, APAP, to enhance its transdermal delivery. This last report also described AOC derivatives that incorporated methoxy ethyleneoxy (EO) or methoxy propyleneoxy (PO) into the alkyl portion of the promoiety in much the same way that Bonina *et al*. [14] incorporated polyethylene glycols into the alkyl portion of carboxylic acid esters promoieties. The more water soluble derivative gave the greater increase in flux of total species containing APAP especially compared to simple AOC derivatives of similar molecular weight.

In order to increase the flexibility of the prodrug approach, we have recently reported the synthesis, solubilities and abilities of several soft alkyl derivatives of APAP to enhance its transdermal delivery [15,16,17]. Soft alkyl derivatives [18] are generally comprised of an acyl enabling functional group [19] which upon hydrolysis results in the formation of an α-hydroxyalkyloxyphenyl intermediate which spontaneously decomposes into the phenol. The soft alkyl derivative approach is not as limited as the simple acyl derivative in terms of possible combinations of functional groups and stability towards chemical hydrolysis: an important consideration for stable formulation development.

Here we report the synthesis, characterization and investigation of the abilities of alkyloxycarbonyl-oxymethyl (AOCOM, **2-4**) and *N*-alkyl-*N*-alkyloxycarbonylaminomethyl (NANAOCAM, **5**) soft alkyl derivatives of APAP that incorporate 2-methoxyethyleneoxy (EO) and 2-methoxy-1-methylethyleneoxy (PO) type groups into the alkyl side chain to enhance the transdermal delivery of APAP. We also compare their performance with their simple straight alkyl chain analogues of similar molecular weights.

## 2. Results and Discussion

### 2.1. Synthesis and physicochemical properties of the AOCOM and NANAOCAM prodrugs

The AOCOM [20] and NANAOCAM [21] prodrugs were synthesized as previously described except that the alkyloxy groups were replaced with 2-methoxyethyleneoxy (EO), 8-methoxytriethyleneoxy (TEO) or 2-methoxy-1-methylethyleneoxy groups (PO). 

**Scheme 1:**
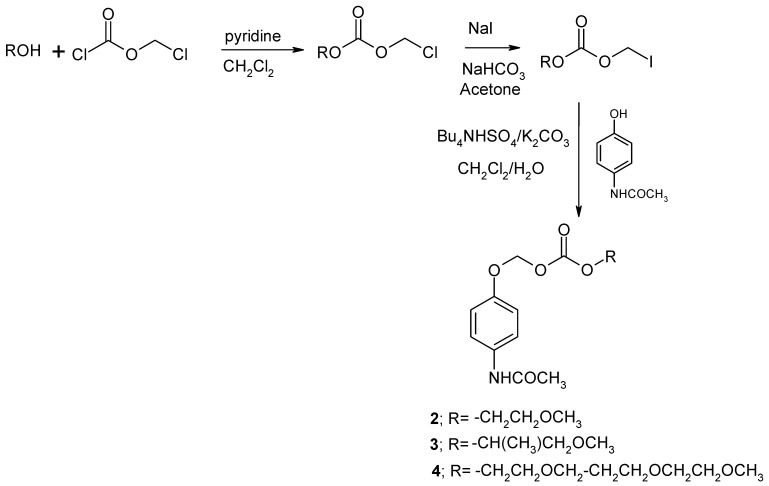
Synthesis of AOCOM.

**Scheme 2:**
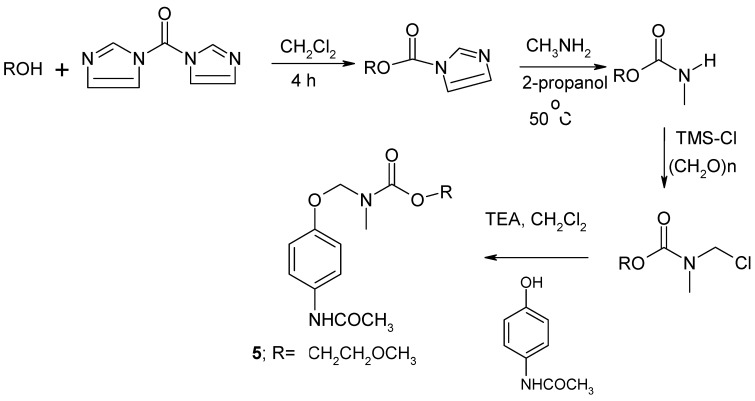
Synthesis of NANAOCAM.

Scheme 1 and Scheme 2 show the general procedure for their syntheses. No attempt was made to optimize the yields, although **3** and **5** were obtained in reasonably good yields from APAP. Only four compounds were made and characterized to test the hypothesis that incorporation of EO, PO or TEO groups would be useful in this application.

Solubilities in isopropyl myristate (S_IPM_) and water (S_AQ_) and partition coefficients between IPM and pH 4.0 buffer (K_IPM:4.0_) were determined for **2** to **5** and are shown in Table 1. The S.D. of the measured S_IPM_ and S_AQ_ were all less than ±5%. The S.D. of the measured K_IPM:4.0_ were less than ±10%. The estimated solubilities in pH 4.0 buffer (S_4.0_) from (S_IPM_)/(K_IPM:4.0_) for **3** and **2** were +29% and –8% of their measured S_AQ_, but **4** was +240%. The overestimation of S_4.0_ compared to S_AQ_ had previously been observed for the more lipophilic members of a series [16], but this was the first time it was observed for the more water soluble member of a series. 

The lipid solubilities of the EO, PO and TEO (collectively EO type derivatives) AOCOM and EO NANAOCAM derivatives were from 2 to 7 times greater than that of APAP. However only **4** had a greater solubility in water than APAP: 1.8 times. Although it is difficult to directly compare these EO type AOCOM derivatives with previous, non-EO type series, when we use the respective molecular weights as the basis, TEO **4** (MW 371) is much less lipid soluble than the non-EO **8** (MW 365, about 10 times) but much more water soluble (3 × 10^4^ times). Similarly, although the molecular weights are not as well matched, EO **2** (MW 283) is much less lipid soluble than the non-EO **7** (MW 267, 4 times) but much more water soluble (15 times). The EO NANAOCAM **5** (MW 296) is also much less soluble in lipid than the non-EO **11** (MW 294, 17 times) but much more water soluble (20 times). Thus, the EO type derivatives are effective at increasing the S_AQ_ of AOCOM and NANAOCAM derivatives using a molecular weight basis for comparison with simple alkyl derivatives but the S_IPM_ values are less. This result may have a negative effect on transdermal delivery since a balance of lipid (S_IPM_) and S_AQ_ is important for optimizing delivery [2,4].

Within the EO type AOCOM series there are three trends that are worth noting. First, when comparing a 2-methoxyethyleneoxy derivative, **2**, with a 2-methoxy-1-methylethyleneoxy derivative, **3**, the S_AQ_ of the derivative with a methyl on the ethyleneoxy group (**3**) is less. A similar result was observed in a comparison of the AOC derivatives **12** and **13**. In each case (**2** versus **3**, and **12** versus **13**) the melting point increased and K_IPM:4.0_ increased with the extra methyl. The extra methyl group led to decreased S_AQ_ but not much increase in S_IPM_ for **2** versus **3**. Second, S_AQ_ can be increased significantly by adding several ethyleneoxy groups to the promoiety. The downside is that there is a significant increase in molecular weight of the prodrug which is counterproductive (see below). Third, the substitution of EO type groups for simple alkyl groups in the promoiety leads to a decrease in partition coefficients. This is the opposite of the expected increase in partition coefficients usually observed for derivatives with higher alkyl carbon content. For instance, TEO AOCOM **4** (MW 371) has a K_IPM:4.0_ value of 0.027 while non-EO AOCOM **8** (MW 365) has a K_IPM:4.0_ value of 26500. Similarly EO NANAOCAM **5** (MW 296) has a K_IPM:4.0_ value of 0.063 while non-EO NANAOCAM **11** (MW 294) has a K_IPM:4.0_ value of 18.6 (Table 2).

### 2.2. Diffusion cell experiments

The diffusion cell experiments were all run using an excess of the permeant in the donor phase so that a saturated solution of the permeant was in equilibrium with the surface of the membrane at steady-state. In this way the prodrugs were all tested at the same thermodynamic activity and the flux values based on delivery of total species containing APAP were all maximum flux values, J_MMIPM_. The J_MMIPM_ values for **2** to **5** are given in Table 2. The S.D. for all J_MMIPM_ values are within ±30% variation in J_M_ values usually observed for *in vitro* experiments using hairless mouse skin as the membrane and IPM as the vehicle [13]. Prodrugs **2** and **5** performed better than APAP, albeit marginally: 1.41 and 1.36 times, respectively.

Again, using MW as the basis for comparison of the delivery of total species containing APAP, J_MMIPM_, EO **2** (MW 283) was about 2.6 times greater than that obtained from non-EO **7** (MW 267). EO **2** was the more water soluble (15 times) but non-EO **7** the more lipid soluble (4.4 times). Similarly, J_MMIPM_ by EO **5** (MW 296) was about 1.12 times greater than that obtained from non-EO **11** (MW 294). EO **5** was the more water soluble (16 times) but non-EO **11** the more lipid soluble (18 times). In both comparisons the more water soluble member gave the greater J_MMIPM_, albeit only marginally in the comparison of **5** and **11** because of the proportional increase in lipid solubility of **11**. On the other hand, in the comparison of TEO **4** (MW 371) with non-EO **8** (MW 365) the more water soluble TEO **4** (3 × 10^4^ times) gave a vastly larger (51 times) J_MMIPM_.

The relatively low J_MMIPM_ values for TEO **4** compared to that of APAP itself and other, less water soluble, members of the AOCOM series on initial examination was disappointing. However, the Roberts-Sloan [4] and other models [22] all contain a strong negative effect of MW on flux. Thus, the relatively low J_MMIPM_ from TEO **4** can be attributed, at least partially, to its relatively high MW. Also, this result suggests that the substitution of EO type groups for alkyl groups in a promoiety to increase S_AQ_ is limited by the rather large increases in MW necessary to significantly increase S_AQ_ (see below).

The % of prodrug converted to APAP during the permeation of mouse skin by the EO type prodrugs was similar to that observed for the non-EO prodrugs: 38–65% for the EO type AOCOM prodrugs versus 54–100% for the non-EO AOCOM prodrugs and 10% for the EO NANAOCAM prodrug versus 22–24% for the non-EO NANAOCAM prodrugs. The difference in the % conversion of AOCOM versus NANAOCAM prodrugs to APAP was thus expected. The relative lower conversion of NANAOCAM prodrugs is due to the stability of NANAOCAM prodrugs to an S_N_1 hydrolysis mechanism where the phenol functions as a leaving group and not to nucleophilic attack by water or an enzyme on the O–(C=O)N carbonyl [15].

The most recent coefficients generated for the fit of log S_IPM_, log S_AQ_ and MW as the independent variables and log J_MMIPM_ as the dependent variables to the Roberts-Sloan (**RS**) equation gave equation 4 for n = 71 [16]:

log J_MMIPM_ = – 0.562 + 0.501 log S_IPM_ + 0.499 log S_AQ_ – 0.00248 MW

Using those coefficients for log S_IPM_, log S_AQ_ and MW, log J_MMIPM_ were predicted for **2** to **5**, and the values for the differences between the log experimental (EXP) J_MMIPM_ and the log predicted (PRE) J_MMIPM_ are given in Table 2. The average of the absolute values for log EXP J_MMIPM_ – log PRE J_MMIPM_ (∆ log J_MMIPM_) was 0.226 ± 0.257 log units (n = 4). However, the major contributor to the poor fit of **2** to **5** was **4**. When **4** was excluded, ∆ log J_MMIPM_ was only 0.097 ± 0.029 log units which is much less than the average of the absolute values of log EXP J_MMIPM_ – log calculated (CALC) J_MMIPM_ for the entire n = 71 database: ∆ log J_MMIPM_ = 0.157 log units. Thus the EO type prodrugs fit as well as non-EO prodrugs to the **RS** equation if **4** is excluded because of the increase in effective MW caused by the associated water molecules (see below). 

Since TEO **4** is much larger than the other prodrugs, the poor fit of **4** may be due to the **RS** equation not having a sufficiently large coefficient for MW. However log EXP – PRE J_MMIPM_ for non-EO **8**, which has a similar MW, was only 0.10 log units, so that does not seem to be an adequate explanation. On the other hand, it is well known that EO groups bind at least two but most likely six water molecules [23] per ethyleneoxy group. Thus, as **4** diffuses through the skin and picks up water molecules, if **4** diffuses with those tightly associated 6 or 18 water molecules, the molecular weight becomes 479 or 695, further exacerbating the negative effect of MW on flux, and log EXP J_MMIPM_ – log PRE J_MMIPM_ for **4** becomes only –0.34 or +0.19 log units, respectively. The effect of the C = O attached to one of the oxygens of the ethylenoxy group is not known, but the electron withdrawing effect of the C = O must surely destabilize the association of water with that oxygen by making the oxygen less basic. Thus, the effect of water associated with a single ethyleneoxy as in **2**, **3**, **5**, **12** and **13** on the MW of the diffusing species must be much less.

## 3. Experimental

### 3.1. General

Melting points were determined on a Meltemp capillary melting point apparatus and are uncorrected. ^1^H NMR spectra were run on a Varian Unity 400 MHz spectrometer. Ultraviolet (UV) spectra were obtained on a Shimadzu UV – 2501 PC spectrophotometer. The vertical Franz diffusion cells (surface area 4.9 cm^2^, 20 ml receptor phase volume, 15 ml donor phase volume) were purchased from Crown Glass (Somerville, NJ, USA). A Fisher (Pittsburgh, PA, USA) circulating water bath was used to maintain a constant temperature of 32 °C in the receptor phase of the diffusion cells. Isopropyl myristate (IPM) was purchased from Givaudan (Clifton, NJ, USA). Theophylline (Th), 2-methoxyethanol, 1-methyl-2-methoxyethanol and triethylene glycol monomethyl ether were purchased from Sigma Chemical Co. (St. Louis, MO, USA); all other chemicals were purchased from Fisher. The female hairless mice (SKH-hr-1) were obtained from Charles River (Boston, MA, USA). All procedures involving the care and experimental treatment of animals were in agreement with the NIH “Principles of Laboratory Animal Care.”

### 3.2. Syntheses

#### 3.2.1. Procedure for the synthesis of the AOCOM prodrugs **2** to **4** [20]

(a) Synthesis of AOCOM iodide: To an ice-cold solution of chloromethyl chloroformate (82.8 mmol and alcohol (69 mmol) in methylene chloride (130 mL) was added pyridine (82.8 mmol) in methylene chloride dropwise over 10 minutes. The mixture was allowed to warm to room temperature and was stirred overnight. The reaction mixture was washed with 1 M HCl (35 mL) and water (35 mL), dried over Na_2_SO_4_, filtered, and concentrated using a rotary evaporator to give the AOCOM chloride as a pale yellow oil. The AOCOM chloride was subsequently dissolved in dry acetone (70 mL) and NaI (91.7 mmol, 1.5 equiv.) was added. This was immediately followed by the addition of NaHCO_3_ (6.1 mmol, 0.1 equiv.) and the resulting mixture was allowed to react at 40 °C for 4 hours. The mixture was concentrated using a rotary evaporator and triturated in methylene chloride for approximately 30 minutes. The resulting mixture was filtered and concentrated as before to give AOCOM iodide as a dark oil.

(b) Alkylation of APAP with AOCOM iodide: A mixture of APAP (28.4 mmol) and K_2_CO_3_ (85.2 mmol) in 140 mL water was allowed to stir several minutes before adding tetrabutylammonium hydrogen sulfate (28.4 mmol) and methylene chloride (70 mL). After several minutes of stirring, a solution of AOCOM iodide (36.8 mmol) in methylene chloride (70 mL) was added in portions to the reaction mixture. The resulting biphasic system was allowed to stir vigorously overnight. The phases were separated and the water layer was extracted with methylene chloride. The organic phases were combined and concentrated under vacuum to give an oily residue. In all cases, TLC of the water phase showed no evidence of unreacted APAP. The residue was then triturated in ether and tetrabutylammonium iodide was removed by vacuum filtration. The prodrugs were purified by column chromatography on silica gel and recrystallized from various solvents to obtain pure samples as described below.

*4-(3’-Oxybutyloxycarbonyloxymethyleneoxy)acetanilide* (**2**). Obtained using 2-methoxyethanol. Purified after silica gel column chromatography eluting with acetone:hexane (20:80 to 35:65) to yield an oil which solidified on standing. The solid was recrystallized from CH_2_Cl_2_:ethyl ether:petroleum ether to give colorless crystals. Yield = 11%, mp = 69-70 °C; ^1^H-NMR (400 MHz, CDCl_3_) δ 7.42 (d, *J* = 9 Hz, 2 H), δ 7.09 (brs, 1 H), δ 7.03 (d, *J* = 9 Hz, 2H), δ 5.74 (s, 2 H), δ 4.33 (m, 2 H), δ 3.63 (m, 2 H), δ 3.39 (s, 3 H), δ 2.17 (s, 3 H); Anal. Calcd. for C_13_H_17_NO_6_: C, 55.12; H, 6.05; N, 4.94. Found: C, 54.95; H, 6.11; N, 4.98.

*4-(3’-Oxy-1’-methylbutyloxycarbonyloxymethyleneoxy)acetanilide* (**3**). Obtained using 1-methyl-2-methoxyethanol. Purified after silica gel column chromatography eluting with acetone:hexane (20:80 to 30:70) to yield an oil which solidified on standing. The solid was recrystallized from CH_2_Cl_2_:ethyl ether:petroleum ether to give colorless crystals. Yield = 62%, mp = 80-82 °C; ^1^H-NMR (400 MHz, CDCl_3_) δ 7.41 (d, *J* = 9 Hz, 2 H), δ 7.18 (brs, 1 H), δ 7.03 (d, *J* = 9 Hz, 2H), δ 5.72 (dd, *J* = 0.8 Hz, 22 Hz, 2 H), δ 4.99 (m, 1 H), δ 3.45 (m, 2 H), δ 3.36 (s, 3 H), δ 2.16 (s, 3 H), δ 1.29 (d, *J* = 6 Hz, 3 H); Anal. Calcd for C_14_H_19_NO_6_: C, 56.56; H, 6.44; N, 4.71. Found: C, 56.69; H, 6.45; N, 4.71.

*4-(3’,6’,9’-Trioxydecyloxycarbonyloxymethyleneoxy)acetanilide* (**4**). Obtained using triethylene glycol monomethyl ether. Purified by silica gel column chromatography eluting with ethyl acetate:hexane (50:50 to 90:10) followed by another column chromatography purification with acetone:hexane (30:70 to 40:60) as the eluent to yield a colorless oil. Yield = 20%, ^1^H-NMR (400 MHz, CDCl_3_) δ 7.43 (d, *J* = 9 Hz, 2 H), δ 7.30 (brs, 1 H), δ 7.01 (d, *J* = 9 Hz, 2 H), δ 5.73 (s, 2 H), δ 4.33 (m, 2 H), δ 3.72 (m, 2 H), δ3.63 (m, 6 H), δ 3.54 (m, 2 H), δ 3.37 (s, 3 H), δ 2.16 (s, 3 H); Anal. Calcd for C_16_H_25_NO_8_: C, 54.98; H, 6.79; N, 3.77. Found: C, 54.27; H, 6.95; N, 3.75.

#### 3.2.2. Procedure for the synthesis of the NANAOCOM prodrug **5** [15]

(a) Methoxyethyloxycarbonylimidazole synthesis: 2-Methoxyethanol (0.01 mol) was reacted with 1,1’-carbonyldiimidazole (0.011 mol, 1.1 equivalents) in CH_2_Cl_2_ (50 mL) overnight at room temperature. The clear solution was diluted with CH_2_Cl_2_ (50 mL), washed with 1N HCl (10 mL) and water (2 × 10 mL). The CH_2_Cl_2_ layer was dried over Na_2_SO_4_ then concentrated to give methoxyethyloxycarbonylimidazole as an oil. Yield = 98%, ^1^H-0NMR (CDCl_3_): δ 3.4 (s, 3H), δ 3.73 (t, 2H), δ 4.55 (t, 2H), δ 7.08 (d, 1H), δ 7.45 (d, 1H), δ 8.15 (s, 1H).

(b) N-Methyl carbamic acid methoxyethyloxy ester: Methoxyethyloxycarbonylimidazole (0.01 mol) was coupled with aqueous methylamine (0.013 mol, 1.3 equivalents) in 2-propanol (10 mL) at 50 °C overnight. The reaction mixture was then concentrated using a rotavapor at 50 °C. The oily residue obtained was dissolved in CH_2_Cl_2_ (50 mL) and washed with 1N HCl (10 mL) and water (5 × 3 mL). The CH_2_Cl_2_ layer was dried over Na_2_SO_4_ and concentrated to give *N*-methyl carbamic acid methoxyethyloxy ester as an oil: yield = 72%, ^1^H-NMR (CDCl_3_): δ 2.8 (d, 3H), δ 3.39 (s, 3H), δ 3.63 (t, 2H), δ 4.24 (t, 2H), δ 4.7 (s, 1H).

(c) N-Methyl-N-methoxyethyloxycarbonylaminomethyl chloride (NANAOCAM-Cl): A suspension of *N*-methyl carbamic acid methoxyethyloxy ester (16 mmol), paraformaldehyde (1.7 eqvs.) and trimethylsilyl chloride (13 eqvs.) was refluxed with a CaCl_2_ drying tube on top of a water condenser, for 2.5 h over an oil bath. The suspension was diluted with CH_2_Cl_2_ and filtered to remove the unreacted paraformaldehyde. The clear filtrate was concentrated using a rotavapor at 40 °C under reduced pressure. The yellow oil obtained was triturated with hexane overnight. The white suspension obtained was filtered, and the filtrate was concentrated to give the desired chloride: yield = 83%, ^1^H- NMR (CDCl_3_): δ 3.02 (s, 3H), δ 3.38 (s, 3H), δ 3.62 (t, 2H), 4.31 (t, 2H), δ 5.33 (s, 2H).

(d) Alkylation of APAP with NANAOCAM-Cl: Equimolar amounts of APAP and triethylamine were refluxed for an hour in CH_2_Cl_2_ followed by the addition of NANAOCAM-Cl (1.1 equivalents) The contents were stirred overnight. The reaction mixture was washed with water. The CH_2_Cl_2_ solution was dried over Na_2_SO_4_ for an hour and filtered. The solution was concentrated using a rotavapor at 40 °C. The resulting material was purified by column chromatography eluting with ethyl acetate:hexane (7:3) followed by trituration in hexane overnight to give *4-(N-methyl-N-3’-oxybutyloxy-carbonylaminomethyleneoxy)acetanilide* (**5**) as a colorless oil. Yield = 65%, R_f_ = 0.14 (7:3, ethyl acetate-hexane); ^1^H-NMR (CDCl_3_): δ 7.3 (d, 2H), δ 7.23 (s, 1H), 6.92-6.96 (2d, 2H), δ 5.26-5.28 (2s, 2H), δ 4.26 (m, 2H), 3.56-3.6 (m, 2H), 3.37 (s, 3H), δ 3.01 (s, 3H), δ 2.15 (s, 3H); Anal. Calcd for C_14_H_20_N_2_O_5_: C, 56.75; H, 6.80; N, 9.45. Found: C, 56.27; H, 6.95; N, 9.75.

### 3.3. Physicochemical properties and analysis

The molar absorptivity of each prodrug at 240 nm (ε_240_) in acetonitrile was determined in triplicate by dissolving a known amount of prodrug in acetonitrile, and analyzing the dilute solution by UV spectro-photometry: AOCOM ε_240_ 1.50 ± 0.045 × 10^4^ L mol^-1^; NANAOCAM ε_240_ 1.44 ± 0.019 × 10^4^ L mol^-1^. For each solid prodrug, the solubility in isopropyl myristate, S_IPM_, was determined in triplicate as previously describes [24] by crushing a sample of the prodrug into a fine powder. Excess powder was added to a test tube containing 3 mL IPM. The test tube was then insulated and the suspension was allowed to stir at room temperature (23 ± 1 °C) for 24 hours on a magnetic stir plate. The suspension was filtered through a 0.25 µm nylon syringe filter. A sample of the filtrate was diluted with acetonitrile and analyzed by UV spectrophotometry. For the prodrug oils, oil was added drop wise to well-stirred IPM until two phases were observed. Another drop of oil was added and stirring continued for 24 hours. The biphasic solution was centrifuged and the IPM layer was removed with a pipette, diluted with acetonitrile and analyzed by UV.

Solubilities in water, S_AQ_, were also determined in triplicate using an identical protocol to the one described above, except that the suspensions or biphasic solutions were only stirred for one hour before filtering. This was done in order to make direct comparisons between the present investigation and previous studies [13,15,16]. In each case, a sample of the filtrate was diluted with acetonitrile and analyzed by UV spectrophotometry.

Partition coefficients were also determined in triplicate for each prodrug by using the saturated IPM solutions obtained from the solubility determinations. An aliquot of the saturated IPM solution was partitioned against pH 4.0 buffer to be more easily compared with previous studies, as in the determination of S_AQ_ (see above), using the following volume ratios (V_4.0_ / V_IPM_) for compounds **2**, **3**, **4** and **5**: 0.5, 0.3, 0.07 and 0.07, respectively. The two phases were vigorously shaken for 10 seconds, which has been shown previously [24] to be sufficient time to give reproducible phase transfer, then allowed to separate via centrifugation. An aliquot of the IPM layer was immediately removed, diluted with acetonitrile, and analyzed by UV spectrophotometry as described above. The partition coefficient was calculated as follows:

K_IPM:4.0_ = [A_a_/(A_b_ – A_a_)]V_4.0_/V_IPM_

where A_b_ and A_a_ are the respective absorbances before and after partitioning, and V_4.0_ and V_IPM_ are the respective volumes of buffer and IPM in each phase. CLogP values were calculated with CHEM DRAW 11.0.

UV spectrophotometry was also used to determine the amount of **1** (APAP) and prodrug present in the receptor phases of the diffusion cells as previously described by Wasdo and Sloan [13], Majumdar and Sloan [15], Thomas and Sloan [16] and Thomas and Sloan [17]. The molar absorptivities of compounds **1** to **5** were determined in pH 7.1 phosphate buffer (0.05 M, I = 0.11 M) containing 0.11% formaldehyde by first dissolving a known amount of either compound in acetonitrile (n = 5). An aliquot (1 mL) of the acetonitrile solution was removed, diluted with buffer, and analyzed by UV spectrophotometry to obtain the molar absorptivities in buffer: APAP ε_240_ 1.01 ± 0.053 × 10^4^ L mol^-1^ and ε_280_ 0.174 ± 0.020 × 10^4^ L mol^-1^; AOCOM **2**-**4** ε_240_ 1.50 ± 0.045 × 10^4^ L mol^-1^ and ε_280_ 0.101 ± 0.014 × 10^4^ L mol^-1^; NANAOCAM **5** ε_240_ 1.44 ± 0.019 × 10^4^ L mol^-1^ and ε_280_ 0.130 ± 0.019 ×10^4^ L mol^-1^. There is considerable overlap between the UV spectra of APAP and its AOCOM prodrugs **2** to **5** so the relative concentrations of each were determined using the following approach. The differences in absorption were found to be greatest at 240 nm and at 280 nm. Therefore, considering the additive nature of absorption, the absorbance at each wavelength (assuming constant cell length) is:

A_240_ = ε_P240_C_P_ + ε_A240_C_A_

A_280_ = ε_P280_C_P_ + ε_A280_C_A_

where A is the absorbance at the respective wavelengths, ε is the molar absorptivity of either the prodrug (P) or APAP (A) at the respective wavelengths, and C is the concentration of the respective compounds in the mixture. Solving the two simultaneous equations gives the following solution for the prodrug concentration C_P_:

C_P_ = (ε_A280_A_240_ – ε_A240_A_280_) / (ε_A280_ε_P240_ – ε_A240_ε_P280_)

Once C_P_ is known, it may be inserted into equation 3 to give the following solution for the concentration of APAP C_A_:

C_A_ = (A_240_ – ε_P240_C_P_) / ε_A240_

### 3.4. Diffusion cell experiments

The maximum flux of each prodrug was measured according to a previously described procedure [25] using skin samples from three different mice. Prior to skin removal, the mice were rendered unconscious by CO_2_, then sacrificed via cervical dislocation. Skins were removed by blunt dissection and placed dermal side down in contact with pH 7.1 phosphate buffer (0.05 M, I = 0.11 M, 32 °C) containing 0.11% formaldehyde (2.7 mL of 37% aqueous formaldehyde/liter) to inhibit microbial growth and maintain the integrity of the skins [26] throughout the experiment. Thus, there was no significant difference in flux from an application of a standard permeant (theophylline, Th) in a standard vehicle (propylene glycol, PG) 4, 24, 48 or 120 hours after the mice were sacrificed and their skins were placed in contact with the receptor phase: 0.0061 ± 0.00056 µmol cm^-2^ h^-1^ at 4 hours, 0.0083 ± 0.0019 µmol cm^-2^ h^-1^ at 24 hours, 0.0094 ± 0.0012 µmol cm^-2^ h^-1^ at 48 hours and 0.010 ± 0.0012 µmol cm^-2^ h^-1^ at 120 hours [26]. A rubber O-ring was placed on top of the skin to ensure a tight seal, and the donor and receiver compartments were fastened together with a metal clamp.

Prior to the application of the prodrug, the skins were kept in contact with buffer for 48 hours to allow any UV absorbing material to leach out and enable subsequent quantification of permeants by UV. During this time, the receptor phase was removed and replaced with buffer 3 times in order to facilitate the leaching process. Twenty four hours before application of the prodrug, a suspension (0.095 M to 0.664 M, i.e. generally 10 × S_IPM_) of the prodrug in IPM was prepared and allowed to mix until it was needed in the diffusion cell experiments. After the 48 hour leaching period, an aliquot (0.5 mL) of the prodrug suspension was added to the surface of the skin (donor phase) as the first application. Samples of the receptor phase were usually taken at 8, 19, 22, 25, 28, 31, 34, and 48 hours and analyzed within 1 hour by UV spectrophotometry as above to determine the amounts of permeated APAP and prodrug. At each sampling time, the entire receptor phase was replaced with fresh buffer in order to maintain sink conditions.

After the 48 hours of the first application period, the donor suspension was removed and the skins were washed three times with methanol (3-5 mL) to remove any residual prodrug from the surface of the skin. The skins were kept in contact with buffer for an additional 24 hours to allow all APAP species (i.e. APAP and prodrug) to leach from the skin. Following this second leaching period, the receptor phase was replaced with fresh buffer and an aliquot (0.5 mL) of a standard drug/vehicle (theophylline/propylene glycol, Th/PG, 200 mg/3 mL) was applied to the skin surface as the second application to serve as a control for determining the integrity of each skin [25] after the first application. Samples of the receptor phase were taken at 1, 2, 3, and 4 hours and analyzed by UV spectrophotometry. No absorption at 240 nm due to APAP or its prodrugs was observed which suggested that 24 hours was sufficient to leach all APAP and its prodrugs from the skins before the second application. The concentration of theophylline in the receptor phase was determined by measuring its absorbance at 270 nm (ε = 10,200 L mol^-1^). At each sampling time, the entire receptor phase was removed and replaced with fresh buffer. The average ± S.D. for these second application fluxes for **2** to **5** (1.04 ± 0.22 µmol cm^-2^ h^-1^) were not significantly different from the average ± S.D. previously reported for these second application fluxes for **6** to **8** (0.983 ± 0.237 µmol cm^-2^ h^-1^) [16], **9** to **11** (0.873 ± 0.355 µmol cm^-2^ h^-1^) [15], **12** and **13** (0.98 and 0.94 µmol cm^-2^ h^-1^, respectively) [13] or the control value for the second application of Th/PG after treatment with IPM alone (1.02 ± 0.13 µmol cm^-2^ h^-1^) [25]. Thus application of IPM or permeant/IPM causes an increase of second application flux compared to no initial application of a permeant in a vehicle (see above) but the increase is reproducible and does not affect the assessment of the relative ability of the prodrug to produce an increase in the delivery of the parent drug by the prodrug [27].

In each experiment, the flux was determined by plotting the cumulative amount of APAP species (APAP plus prodrug) against time. Flux could then be calculated by dividing the slope of the steady-state portion of the graph (19 to 34 hours with 5 data points, r^2^ > 0.99 for each plot, although there was no decrease in the incremental increase of cumulative amount at 48 hours) by the surface area of the skin (4.9 cm^2^).

## 4. Conclusions

The incorporation of EO type groups into the promoieties of AOCOM and NANAOCAM prodrugs of APAP do not increase the S_AQ_ values of the prodrugs above that of APAP unless more than one EO is incorporated. Although the best EO type (EO, PO and TEO) soft alkylated prodrugs increased J_MMIPM_, it was not sufficient to warrant taking the extra steps to synthesize them compared to the non-EO type prodrugs for this application.

The J_MMIPM_ values of the EO type soft alkyl prodrugs was well predicted by the Roberts-Sloan equation. Thus, incorporation of oxygen into the alkyl chain of the promoiety of homologous series does not affect the ability of the Roberts-Sloan to predict J_MMIPM_. The effect of S_AQ_ on the design of prodrugs to enhance topical delivery remains as an important consideration.

## Figures and Tables

**Table 1:**
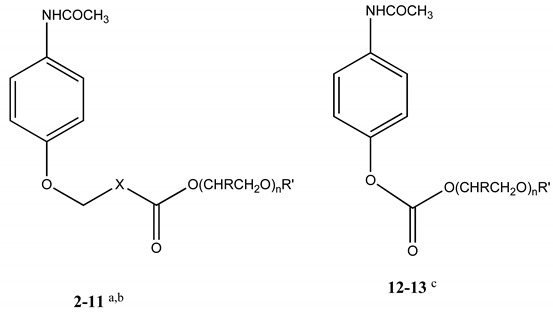
Physicochemical Properties:Molecular Weight (MW), Melting Point (mp), Solubility in IPM (S_IPM_), Water Solubility (S_AQ_) of AOCOM, NANAOCAM and AOC Prodrugs of APAP.

Compound	X	n	R	R’	MW	mp ^o^C	S_IPM_*^d^*	S_AQ_*^d^*
APAP, **1**					151	167-170	1.9	100
**2**	O	1	H	CH_3_	283	69-70	10.43	31.1
**3**	O	1	CH_3_	CH_3_	297	80-82	14.01	7.68
**4**	O	3	H	CH_3_	371	oil	12.14	184.0
**5**	N(CH_3_)	1	H	CH_3_	296	oil	4.16^e^	66.1
**6**	O	0	-	C_2_H_5_	253	83-85	20.7	7.76
**7**	O	0	-	C_3_H_7_	267	68-69	45.8	2.00
**8**	O	0	-	C_10_H_21_	365	54-56	130	0.0056
**9**	N(CH_3_)	0	-	C_2_H_5_	266	75-77	19.9	15.85
**10**	N(CH_3_)	0	-	C_3_H_7_	280	59-60	40.7	8.91
**11**	N(CH_3_)	0	-	C_4_H_9_	294	oil	67.6	3.63
**12**	-	1	H	CH_3_	253	78-81	10.23	34.67
**13**	-	1	CH_3_	CH_3_	267	120-123	3.38	3.31

*^a^* Data for **6** to **8** from [16]. *^b^* Data for **9** to **11** from [15]. ^c^ Data for **1**, **12**, **13** from [13]. *^d^* Units of mM. ^e^ Estimated from K_IPM:4.0_ and S_AQ_.

**Table 2:** Partition Coefficients Between IPM and pH 4.0 Buffer (K_IPM:4.0_), Flux from IPM Through Hairless Mice Skins (J_MMIPM_), Percentage of Parent Drug Obtained After Permeation Through Hairless Mice Skins of AOCOM, NANAOCAM and AOC prodrugs of APAP and log EXP- log PRE J_MMIPM_.

Compound ^a,b,c^	K_IPM:4.0_	CLogP	EXP J_MMIPM_	% Parent Drug	log EXP J_MMIPM_^d^	log EXP-log PRE J_MMIPM_^e^
**1**	-	0.49	0.51	-	-0.29	-0.49
**2**	0.364	0.93	0.72	38	-0.14	-0.13
**3**	1.41	1.24	0.433	65	-0.36	-0.08
**4**	0.027	0.66	0.379	52	-0.42	-0.61
**5**	0.063	1.51	0.696	10	-0.16	-0.08
**6**	2.76	1.51	0.66	54	-0.18	-0.12
**7**	9.21	1.85	0.283	75	-0.55	-0.31
**8**	26500	5.56	0.00739	100	-2.13	-0.1
**9**	1.20	1.80	0.51	23	-0.29	-0.32
**10**	4.89	2.33	0.43	22	-0.37	-0.27
**11**	18.6	2.86	0.62	24	-0.21	-0.24
**12**	0.50	0.54	0.776	49	-0.11	-0.19
**13**	1.44	0.85	0.087	100	-1.06	-0.36

^a, b, c^ same as Table 1. ^d^ Units of μmole cm^-2^h^-1^. ^e^ The value for log predicted (PRE) J_MMIPM_ from Equation 4.
